# Right-sided subcutaneous implantable cardioverter-defibrillator placement in a patient with “acquired mesocardia”

**DOI:** 10.1016/j.hroo.2022.08.003

**Published:** 2022-10-21

**Authors:** George S. Prousi, Judy Nichols, Sony Jacob

**Affiliations:** ∗ Division of Cardiology, Prisma Health/University of South Carolina, Columbia, South Carolina; † Division of Cardiac Electrophysiology, Columbia Veterans Affairs Medical Center, Columbia, South Carolina

**Keywords:** Mesocardia, Right-sided subcutaneous implantable cardioverter-defibrillator, Anatomic variation, Sudden cardiac death, Sensing vectors

**WHAT WE LEARNED FROM THIS CASE:**

• Patients requiring implantable cardioverter-defibrillator (ICD) therapy without favorable anatomy or with contraindications to transvenous systems and do not require pacing may be candidates for subcutaneous implantable cardioverter-defibrillator (S-ICD) systems.
• S-ICDs can be effectively implanted in nontraditional positions for patients with varying anatomy, including, but not limited to, mesocardia. Modified locations of both the pulse generator and lead should be tested using electrocardiographic (ECG) criteria and imaging to deduce optimal sensing and defibrillation.
• Screening ECGs with modified locations of the ICD and lead should be scrutinized to ensure optimal device placement and should be conducted in various positions and postures before implantation.

Implantable cardioverter-defibrillators (ICDs) are widely used for prevention of sudden cardiac death.^1^ Transvenous ICDs remain the mainstay of therapy; however, limitations and complications exist.^2^ In such cases, subcutaneous implantable cardioverter-defibrillator (S-ICD) systems should be considered, and thought should be given to device and lead positioning.^3^ Right-sided implantation in patients with congenital heart disease (CHD) and dextrocardia have been discussed; however, few data exist regarding non-CHD patients receiving right-sided S-ICD devices.^4^^,^^5^ We describe a case of right-sided S-ICD implantation in a patient with “acquired mesocardia” and discuss its rationale, effectiveness, and clinical implications.

A 63-year-old man with nonischemic cardiomyopathy (ejection fraction 25%) and left hemidiaphragmatic paralysis with resultant “acquired mesocardia” presented for consideration of ICD implantation. Echocardiography confirmed reduced ventricular function, and chest radiography showcased a persistent elevated left hemidiaphragm and mesocardia. No indication for atrial or ventricular pacing was identified, and S-ICD implantation was pursued.

Detailed screening for the S-ICD was performed, and special attention was given to the detection consistency of the QRS and the ability to distinguish T waves, in addition to the electrical shock vector needed to encompass the displaced left ventricular mass. Electrocardiographic screening was conducted with leads positioned on the traditional left side and modified leads on the proposed right side in both supine and erect postures. During the template screening test, right-sided electrocardiographic electrode tracings passed 2 sensing vectors in the erect position and all 3 sensing vectors in the supine position, while traditional left-sided tracings failed. Screenings were performed with various anteroposterior locations on the right side for the pulse generator, and both left and right parasternal locations for the lead.

**Figure 1:**
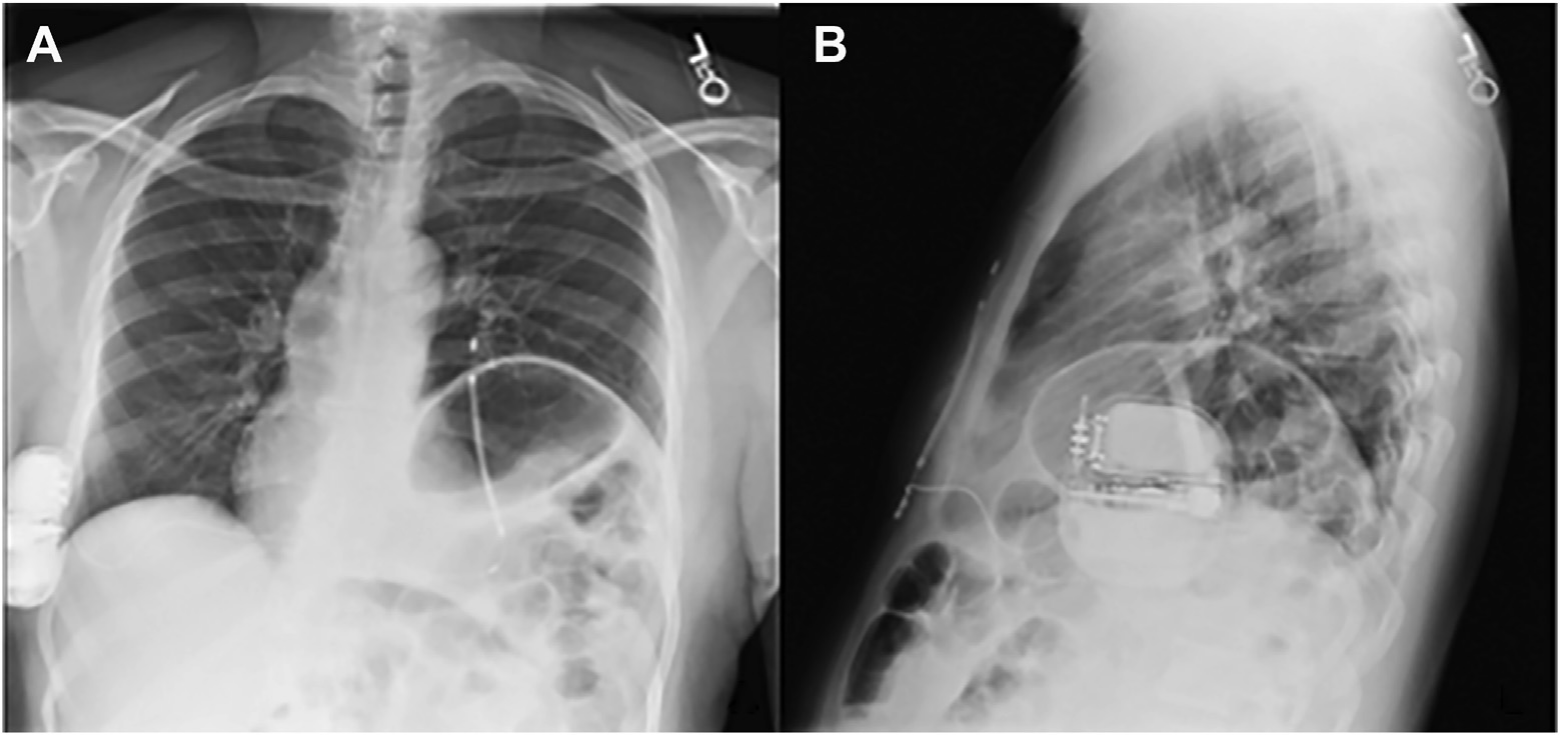
Posteroanterior **(A)** and lateral **(B)** plain chest radiographs showing right, lateral thoracic placement of the pulse generator with corresponding left lateral coil placement of the subcutaneous implantable cardioverter-defibrillator. Note the presence of a prominent left hemidiaphragm and associated mesocardia.

The device was implanted in the right lateral thorax with the coil extending left parasternally using the described screening tests and chest radiography (Figure 1). Importantly, the pulse generator was placed posteriorly along the right chest wall and close to the rib cage (over the muscle fascia with no adipose tissue in between) to optimize the electrical defibrillation vector and minimize sensing error, allowing for the primary vector to lie maximally orthogonal to the cardiac axis. Periprocedural defibrillation threshold testing was successful on the first attempt at 80 J with impedance of 61 Ω.

ICDs remain an effective therapy for the prevention of sudden cardiac death.^6^ However, transvenous systems are associated with complications such as infection, venous occlusion, lead perforation, and pericardial tamponade.^2^ Additionally, anatomic limitations coupled with consideration of only temporary ICD use may prompt the pursuit of device alternatives.

Subcutaneous ICD systems (S-ICD^TM^ System, Boston Scientific, Marlborough, MA) have enhanced clinical practices for circumstances in which transvenous devices either are not suitable or are contraindicated.^7^ Traditionally, S-ICDs and electrodes are placed subcutaneously in the extrathoracic position along the left lateral thorax with coil positioning immediately left parasternal.^8^ This principle of minimizing electrical inertness by avoiding subcutaneous fat tissue has been validated by the PRAETORIAN score but is limited to left-sided S-ICD placement.^9^

To the best of our knowledge, our case represents the first right-sided S-ICD device to be implanted because of “acquired mesocardia” related to left hemidiaphragmatic paralysis. The patient’s nontraditional anatomy presented as a “pseudo-dextrocardia” with left ventricular cardiac mass favoring the right side. Literature has described right-sided S-ICD placement in patients with congenital dextrocardia; however, we propose the potential for more widespread use for other patients, such as those with altered anatomy secondary to pneumonectomy or diaphragmatic trauma, and the growing population of patients with a left ventricular assist device. Additionally, fhe automated sensing algorithms featured in newer-generation S-ICDs may identify potential benefits to alternative device placement. Given our success with right-sided S-ICD implantation, anatomic and nonanatomic variations, if present, should be accounted for but should not be a limiting factor.

In patients with anatomic and nonanatomic circumstances precluding traditional left-sided S-ICD placement, right-sided implantation or other modified locations may be a suitable alternative. Extensive electrical screening and imaging modalities will help identify the optimal location in such case scenarios, allowing for a more tailored approach to S-ICD implantation.
